# Independent and interactive effect of sitting time and physical activity on prevalence of hyperuricemia: the Henan Rural Cohort Study

**DOI:** 10.1186/s13075-020-02385-8

**Published:** 2021-01-06

**Authors:** Xiaokang Dong, Yuqian Li, Lulu Zhang, Xiaotian Liu, Runqi Tu, Yikang Wang, Ruiying Li, Linlin Li, Jian Hou, Zhenxing Mao, Wenqian Huo, Chongjian Wang

**Affiliations:** 1 Department of Epidemiology and Biostatistics, College of Public Health, Zhengzhou University, 100 Kexue Avenue, Zhengzhou, 450001 Henan People’s Republic of China; 2 Department of Clinical Pharmacology, School of Pharmaceutical Science, Zhengzhou University, Zhengzhou, Henan People’s Republic of China; 3 Department of Occupational and Environmental Health Science, College of Public Health, Zhengzhou University, 100 Kexue Avenue, Zhengzhou, Henan 450001 People’s Republic of China

**Keywords:** Physical activity, Sitting time, Hyperuricemia, Interaction

## Abstract

**Background:**

There are few studies on the hyperuricemia (HUA) and moderate to vigorous intensity physical activity (PA) and also hardly regarding sitting time (ST). The purpose of this study was to examine the independent and interactive association of PA and ST with HUA.

**Methods:**

A cross-sectional analysis was performed on 38,855 participants (aged 18–79) enrolled from the Henan Rural Cohort Study at baseline (2015 to 2017). PA and ST levels were assessed by using the International Physical Activity Questionnaire (IPAQ). HUA was defined as a serum uric acid level of > 7.0 mg/dL for males and > 6.0 mg/dL for females. Multivariable logistic regression and linear regression models were applied to examine the independent association between PA or ST and HUA and serum uric acid level. Interaction plots were used to visualize the interaction effects of PA and ST on HUA.

**Results:**

PA level was inversely related with serum uric acid level (*β* − 0.15, 95% confidence interval (*CI*) − 0.22, − 0.07), but ST was positively related with uric acid level (*β* 2.12, 95% CI 1.90, 2.34). Metabolic equivalent (MET-hour/day) was associated with decreased prevalence of HUA (odds ratio (*OR*) 0.97, 95% CI 0.96, 0.99), while per hour increased for ST was associated with increased HUA (OR 1.05, 95% CI 1.04, 1.06). The interaction of PA and ST was significant (*P* < 0.001).

**Conclusion:**

Exposure to higher ST was independently related to increased prevalence of HUA, while vigorous PA with a decreased HUA prevalence. Meanwhile, higher daily ST might attenuate the protective effect of PA on HUA.

**Trial registration:**

The Henan Rural Cohort Study has been registered at Chinese Clinical Trial Register (Registration number: ChiCTR-OOC-15006699).

## Background

Serum uric acid level and the prevalence and incidence of hyperuricemia (HUA) in the world population have been steadily rising over the past four decades [1, 2].

HUA is not only a pre-requisite for gout, but also is associated with the many chronic metabolic diseases such as cardiovascular disease (CVD) [3], diabetes [4], metabolic syndrome [5], and chronic kidney disease [6]. The increasing prevalence of HUA has been considered as an emerging public health concern over the world [1, 2, 7, 8], and the study of the HUA and its risk factors has become even more important.

Lifestyle behaviors play important roles on individual health, such as physical activity (PA) and sedentary behavior [9]. The health effects of regular PA have been well documented, and the lack of PA is well recognized to increase the incidence and mortality of various chronic diseases, including diabetes, stroke, and cardiovascular disease [9–11]. Sedentary behavior, a unique aspect of lifestyle behavior, might not simply represent the extreme low PA level, as many studies have found that prolonged sitting time (ST) is independently linked to human metabolism health given PA level [12]. Thus, both inadequate PA and long ST are necessarily considered as an important public health problem.

Although pervious studies have found there was a negative association between PA and prevalence of HUA [1, 13], little is known about its independent effect on HUA according to multiples of the basal metabolic rate. Moreover, the relationship of ST with HUA also remains unclear. Only a recent study conducted in Korean found that regular PA and reduced sedentary time might reduce the prevalence of HUA [14]. However, the interactive effect of PA and ST on health has not been reported, which is important for the development of guidelines in recent years [15]. Whether PA can offset the adverse effect of ST still needs to be elucidated. In addition, there is a high prevalence of physical inactivity and sedentary behavior [16] as well as prevalent HUA in Chinese rural areas [17]. Given these, it is necessary to conduct this study to examine the independent association of PA and ST with serum uric acid level and HUA in rural Chinese population, and further to estimate the interaction effect of PA and ST on HUA.

## Methods

### Study population

The analyses are based on data from the Henan Rural Cohort Study, which was a prospective study of chronic non-communicable diseases, and the cohort profile and study population have been described in elsewhere [18, 19]. In brief, the study was conducted in Yuzhou, Xinxiang, Tongxu, Yima, and Suiping county of Henan Province, China, from July 2015 to September 2017. A multistage cluster sampling method was applied to recruit individuals aged 18–79 years from the general population. In the first stage, five different geographical regions (south, central, north, east, and west) were selected by simple cluster sampling from Henan province. In the second stage, considering the coherence of the residents, population stability, and local medical conditions, 1–3 typical rural townships of each county were selected by the local Centre for Disease Control and Prevention. In the final stage, permanent residents who signed informed consent were included as the study sample from each rural village of the selected township. Overall, 39,259 eligible individuals completed the baseline survey. In the current study, a total of 404 individuals were excluded from the present analysis due to missing data on serum uric acid level (*n* = 54), and diagnosed with cancer and serious renal disease (*n* = 350). Finally, 38,855 participants (15,371 males and 23,484 females) were included in the current analysis. The study complied with the 1975 Declaration of Helsinki and was approved by the ethics committee of Zhengzhou University. Written informed consent was obtained from each participant before starting this study.

### Definitions of exposure

PA and ST levels were assessed by using the International Physical Activity Questionnaire (IPAQ) [20]. As the details have been described previously [16, 19], PA-metabolic equivalent (MET) was calculated by using MET coefficient of activity × duration (hour per time) × frequency (times per week) [19]. Therefore, according to the categories of PA, each category of vigorous activity, moderate activity, and walking was calculated with the corresponding MET values of 8, 4, and 3.3, respectively. The total value of MET was obtained by adding value of these three categories. Finally, based on the standard scoring criteria of IPAQ, three levels of PA were determined: the light (physically inactive) activity category was for individuals who met neither moderate activity nor vigorous activity criteria, moderate activity should meet either of the following four criteria: (1) moderate activity was at least 30 min in 5 days a week, (2) vigorous activity was at least 20 min in 3 days a week, (3) walking at least 30 min a day for five or more days a week, and (4) the accumulating Mets, of any combination of moderate activity, vigorous activity, or walking, was at least 600 MET-min/week in five or more days a week; and vigorous activity should meet either of the following two criteria: (1) vigorous activity was at least 3 days/week and the week of accumulating Mets was at least 1500 MET-min/week and (2) any combination of vigorous activity, moderate activity, or walking at least 5 days/week, and the accumulating Mets was at least 3000 MET-min/week [16]. The individual total ST every day in the past week was estimated by using the question “About how many hours in each 24-h day do you usually spend on sitting? Same as some recent studies [21], daily ST was classified by four groups: < 4 h/day, 4 to 6 h/day, 6 to 8 h/day, and **≥** 8 h/day.

### Definition of HUA

Blood samples were obtained from each individual after at least 8 h of overnight fasting to measure multiple biochemical indicators. Serum uric acid level was measured by ROCHE Cobas C501 automatic biochemical analyzer with enzymatic colorimetric method. HUA was defined as serum uric acid level > 7.0 mg/dL (417 μmol/L) in males and serum uric acid level > 6.0 mg/dL (357 μmol/L) in females among Chinese population [17].

### Potential covariates

Potential confounders were similar to our previous study of HUA [17], including demographic covariates (age and gender), socioeconomic covariates (marital status, average monthly individual income, and education level), and lifestyle factors (smoking, alcohol drinking, dietary, etc.), which were collected through face-to-face interview by trained research staff using a standardized questionnaire. Socioeconomic covariates included education level (“primary school or below,” “middle school and high school or above”), marital status (“married/living together” or “divorced/widowed/separated/ unmarried”), and average monthly income (“< 500 RMB,” “500–1000 RMB,” or “> 1000 RMB”). Smoking status was classified into current, former, and never groups. Drinking status also was classified into current, former, and never groups. The four-cluster dietary patterns were obtained in the current study by factor analysis using the previous standard principal component analysis method [22]. Body weight and height were measured with the standard methods, and body mass index (BMI) was calculated as weight in kilograms divided by height in meters squared. Blood pressure was measured 3 times on the right arm at heart level sitting position.

### Statistical analysis

The differences in basic characteristics between HUA and non-HUA participants were tested using the Student’s *t* test and the chi-square test. Continuous variables and categorical variables were expressed as mean ± standard deviations (SD) and counts and percentage, respectively.

The associations between PA or ST and serum uric acid level were analyzed using multiple linear regression models and presented as *β* values with 95% confidence intervals (*CI*), where the light PA and the lowest sitting (< 4 h/day) served as the reference categories, respectively. Multivariable-adjusted logistic regression models also applied to assess associations of PA or ST and prevalence of HUA, results are presented as odds ratio (*OR*) with corresponding 95% *CI*. Similarly, the effects of MET-hour/day and per hour increased in ST on serum uric acid level or prevalence of HUA were estimated. All potential covariates were selected based on the existing literature, and two models were performed: model 1 was adjusted for PA and ST level where applicable; model 2 included ST and PA as well as age, gender, education level, marital status, average monthly income, smoking status, drinking status, dietary pattern, and BMI. In addition, a cross-product term was incorporated into logistic regression models to evaluate the statistical significance of interaction of PA and ST on HUA. Restricted cubic spline regression was used to explore the dose–response relationship between continuous PA-MET hour or ST (h/day) and HUA.

Meantime, stratified analyses were performed to estimate the effects of PA on HUA in different ST groups. Joint association between PA and ST also was examined the by deriving a combined variable with 12 groups, where the combined vigorous PA and lowest ST (< 4 h/day) served as the reference group. Furthermore, generalized linear models were also employed to visualize the interactive effect of ST (h/day) and PA (MET-hour/day) on HUA. Effect estimates of MET-hour/day were plotted with their 95% *CI* as a function of increasing ST (h/day) in an interaction plot.

All analyses were conducted using IBM SPSS V.19.0 and R 3.5.0. A two tail of *P* value < 0.05 was considered statistically significance.

## Results

### Descriptive statistics

The basic characteristics of the study participants by HUA status are summarized in Table 1. The mean age of total participants was 55.56 years (*SD* = 12.21 years), and more than half of the subjects were females (60.44%). The mean serum uric acid level was 286.49 μmol/L (*SD* = 79.64 μmol/L), and the prevalence of HUA was 10.24% among total participants. Participants with HUA were younger than Non-HUA population (54.04 vs. 55.74 years), had higher education level and average monthly income, and were more likely to be smoker, drinker, and with low PA and high ST (all *P* < 0.001). Moreover, they also had higher BMI and serum uric acid level (both *P* < 0.001).

**Table 1:** Basic characteristics of study participants by with and without hyperuricemia (*n* = 38,855)

Characteristics	Total (*n* = 38,855)	Non-HUA (*n* = 34,877)	HUA (*n* = 3978)	*P* value
Age (year, mean ± *SD*)	55.56 ± 12.21	55.74 ± 12.05	54.04 ± 13.43	< 0.001^a^
Gender (*n*, %)				< 0.001^b^
Male	15,371 (39.56)	13,403 (38.43)	1968 (49.47)	
Female	23,484 (60.44)	21,474 (61.57)	2010 (50.53)	
Education level (*n*, %)				< 0.001^b^
≤ Primary school	17,385 (44.74)	15,748 (45.15)	1637 (41.15)	
≥ Middle school	21,470 (55.26)	19,129 (54.85)	2341 (58.85)	
Marital status (*n*, %)				0.690 ^b^
Married/living together	34,872 (89.75)	31,309 (89.77)	3563 (89.57)	
Divorced/widowed/separated/unmarried	3983 (10.25)	3568 (10.23)	415 (10.43)	
Average monthly income (*n*, %)				< 0.001^b^
< 500 RMB	13,837 (35.61)	12,432 (35.65)	1405 (35.32)	
500–1000 RMB	12,795 (32.93)	11,608 (33.28)	1187 (29.84)	
> 1000 RMB	12,223 (31.46)	10,837 (31.07)	1386 (34.84)	
Smoking status (*n*, %)				< 0.001^b^
Never	28,260 (72.73)	25,628 (73.48)	2632 (66.16)	
Former	3134 (8.07)	2739 (7.85)	395 (9.93)	
Current	7461 (19.20)	6510 (18.67)	951 (23.91)	
Drinking status (*n*, %)				< 0.001^b^
Never	30,018 (77.26)	27,323 (78.34)	2695 (67.74)	
Former	1796 (4.62)	1610 (4.62)	186 (4.68)	
Current	7041 (18.12)	5944 (17.04)	1097 (27.58)	
Physical activity, *n* (%)				< 0.001^b^
Light	12,563 (32.33)	11,077 (31.76)	1486 (37.36)	
Moderate	14,644 (37.69)	13,222 (37.91)	1422 (35.75)	
Vigorous	11,648 (29.98)	10,578 (30.33)	1070 (26.90)	
MET-Hour/day, (mean ± *SD*)	18.09 ± 10.11	18.09 ± 10.11	16.81 ± 10.14	< 0.001^a^
Sitting time				< 0.001^b^
< 4 h/day	11,863 (30.53)	10,846 (31.10)	1017 (25.57)	
4–6 h/day	10,377 (26.71)	9386 (26.91)	991 (24.91)	
6–8 h/day	6498 (26.04)	5798 (16.62)	700 (17.60)	
≥ 8 h/day	10,117 ()	8847 (25.37)	1270 (31.93)	
Sitting time (h/day, mean ± *SD*)	5.59 ± 3.29	5.53 ± 3.26	6.09 ± 3.50	< 0.001^a^
Dietary pattern				< 0.001^b^
Pattern I	7088 (23.90)	7088 (23.90)	1050 (30.02)	
Pattern II	7820 (26.36)	7820 (26.36)	803 (22.96)	
Pattern III	8362 (28.19)	8362 (28.19)	964 (27.56)	
Pattern IV	6393 (21.55)	6393 (21.55)	681 (19.47)	
BMI (kg/m^2^, mean ± *SD*)	24.84 ± 3.57	24.63 ± 3.49	26.66 ± 3.73	< 0.001^a^
Serum uric acid level (μmol/L, mean ± *SD*)	286.49 ± 79.64	269.20 ± 60.69	438.14 ± 63.57	< 0.001^a^

*SD* standard deviation, *T2DM* type 2 diabetes mellitus

^a^Student’s *t* test was used to compare normal distributed continuous variables among the groups

^b^A chi-square test was used to test the distributions of categorical variables among the groups

### Association of PA or ST with serum uric acid level and HUA

**Fig. 1:**
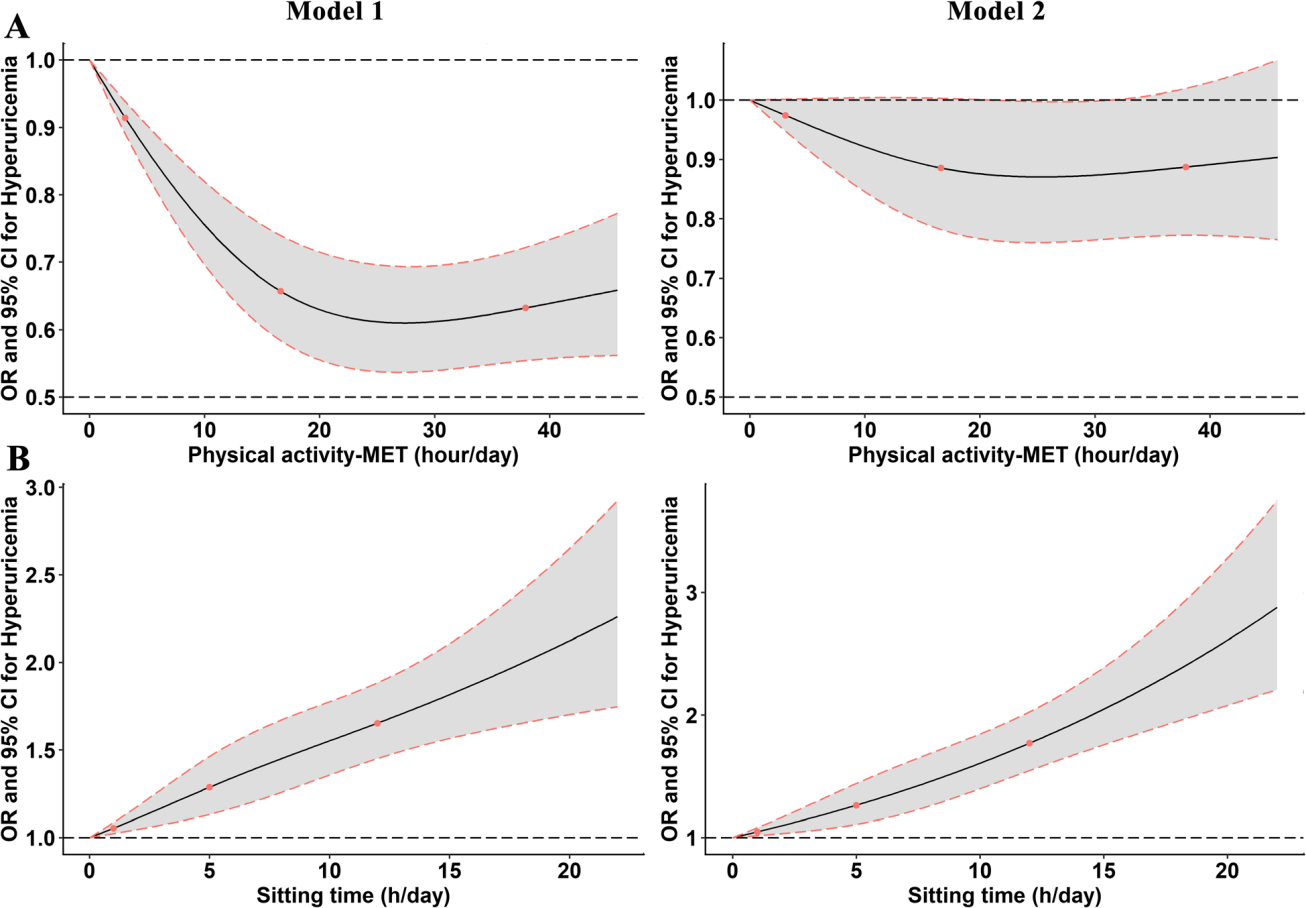
The association between physical activity-MET (hour/day) (**a**) or sitting time (h/day) (**b**) and prevalence of hyperuricemia from restricted cubic splines. Model 1: adjusted by physical activity and sitting time (h/day) where applicable; Model 2: adjusted by physical activity and sitting time (h/day) as well as age, gender, education level, marital status, average monthly income, smoking status, drinking status, dietary pattern, and BMI

Table 2 summarizes the linear relationship between PA level or ST with serum uric acid level. As a whole, the serum uric acid level increased with decreasing PA level or increasing ST. In model 2, compared with light PA or ST of < 4 h/d, the *βs* and 95% *CIs* for moderate and vigorous PA, ST of 4–6, 6–8, and ≥ 8 h/day were 0.76 (− 0.97, 2.48) and − 4.27 (− 6.10, − 2.45), 2.74 (0.89, 4.59), 8.12 (5.99, 10.26), and 17.60 (15.70, 19.51), respectively. The *βs* (95% *CIs*) for MET-hour/day and per hour increased were − 0.15 (− 0.22, − 0.07) (*P* for trend < 0.001) and 2.12 (1.90, 2.34) (*P* for trend < 0.001). In multivariable-adjusted logistic regression analysis, PA level was inversely associated with the prevalence of HUA, while ST was positively associated with prevalence of HUA (Table 3 and Fig. 1). After adjusting for potential covariates in model 2, the *ORs* (95% *CIs*) for HUA comparing vigorous and moderate PA to light group were 0.94 (0.87, 1.02) and 0.84 (0.77, 0.91), respectively (*P* for trend < 0.001); the *ORs* (95% *CI*s) for HUA comparing ST 4–6, 6–8 and ≥ 8 h/d to < 4 h/d were 1.11 (1.01, 1.22), 1.28 (1.15, 1.42), and 1.50 (1.36, 1.64) (*P* for trend < 0.001); when HUA was analyzed per MET-hour/day and per hour for ST increment, the adjusted *ORs* (95% *CIs*) were 0.97 (0.96, 0.99) and 1.05 (1.04, 1.06), respectively (Table 3). In the sex-stratified analyses (Additional file 1: Tables S1 and S2), similar associations were observed in both males and females. Meantime, in Fig. 1, the restricted cubic spline showed that there was a linear dose–response relationship between ST and HUA (*P* for non-linearity > 0.05), and a non-linear dose–response relationship for PA (*P* for non-linearity < 0.05). Finally, when the interaction term was included, a significant interaction effect of ST and PA on HUA was observed (Table 3, *P* < 0.001).

**Table 2:** Association of physical activity level and sitting time with serum uric acid level

	Number	Serum uric acid level (μmol/L, mean ± ***SD***)	***β*** (95% ***CI***)	
			Model 1	Model 2
**Physical activity level**				
**Light**	12,563	293.03 ± 82.57	0 (Ref.)	0 (Ref.)
**Moderate**	14,644	280.70 ± 77.92	− 9.53 (− 11.46, − 7.60)	0.76 (− 0.97, 2.48)
**Vigorous**	11,648	286.73 ± 78.00	− 1.75 (− 3.80, 0.30)	− 4.27 (− 6.10, − 2.45)
**MET-hour/d**			− 0.29 (− 0.37, − 0.21)	− 0.15 (− 0.22, − 0.07)
***P*** **value for trend**			< 0.001	< 0.001
**Sitting time**				
**< 4 h/day**	11,863	278.27 ± 77.77	0 (Ref.)	0 (Ref.)
**4–6 h/d**	10,377	282.30 ± 78.25	4.85 (2.76, 6.94)	2.74 (0.89, 4.59)
**6–8 h/d**	6498	287.84 ± 80.75	10.75 (8.34, 13.16)	8.12 (5.99, 10.26)
**≥ 8 h/day**	10,117	299.57 ± 80.81	20.63 (18.48, 22.77)	17.60 (15.70, 19.51)
**Per hour increased**			2.44 (2.19, 2.68)	2.12 (1.90, 2.34)
***P*** **value for trend**			< 0.001	< 0.001

Multivariable model 1 adjusted for physical activity and sitting time level where applicable; model 2 included sitting time and physical activity as well as age, gender, education level, marital status, average monthly income, smoking status, drinking status, dietary pattern and BMI. *SD*, standard deviation; *CI* confidence interval

**Table 3:** Association of physical activity level and sitting time with prevalence of hyperuricemia

	Cases/number	Prevalence (95% ***CI***)	***OR*** (95%***CI***)	
			Model 1	Model 2
**Physical activity level**				
**Light**	1486/12563	11.83 (11.26, 12.39)	1 (Ref.)	1 (Ref.)
**Moderate**	1422/14644	9.71 (9.23, 10.19)	0.84 (0.78, 0.91)	0.94 (0.87, 1.02)
**Vigorous**	1070/11648	9.19 (8.66, 9.71)	0.82 (0.75, 0.89)	0.84 (0.77, 0.91)
**MET-hour/day**			0.95 (0.93, 0.97)	0.97 (0.96, 0.99)
***P*** **value for trend**			< 0.001	< 0.001
**Sitting time**				
**< 4 h/day**	1017/11863	8.57 (8.07, 9.08)	1 (Ref.)	1 (Ref.)
**4–6 h/day**	991/10377	9.55 (8.98, 10.12)	1.13 (1.03, 1.24)	1.11 (1.01, 1.22)
**6–8 h/day**	700/6498	10.77 (10.02, 11.53)	1.29 (1.16, 1.42)	1.28 (1.15, 1.42)
**≥ 8 h/day**	1270/10117	12.55 (11.91, 13.20)	1.46 (1.34, 1.60)	1.50 (1.36, 1.64)
**Per hour increased**			1.04 (1.03, 1.05)	1.05 (1.04, 1.06)
***P*** **value for trend**			< 0.001	< 0.001
***P***_***Sitting time****×****Physical activity***_			0.006	< 0.001

Multivariable model 1 adjusted for physical activity and sitting time level where applicable; model 2 included sitting time and physical activity as well as age, gender, education level, marital status, average monthly income, smoking status, drinking status, dietary pattern and BMI. *OR* odds ratio, *CI* confidence interval

### Stratified analysis and joint analysis

**Fig. 2:**
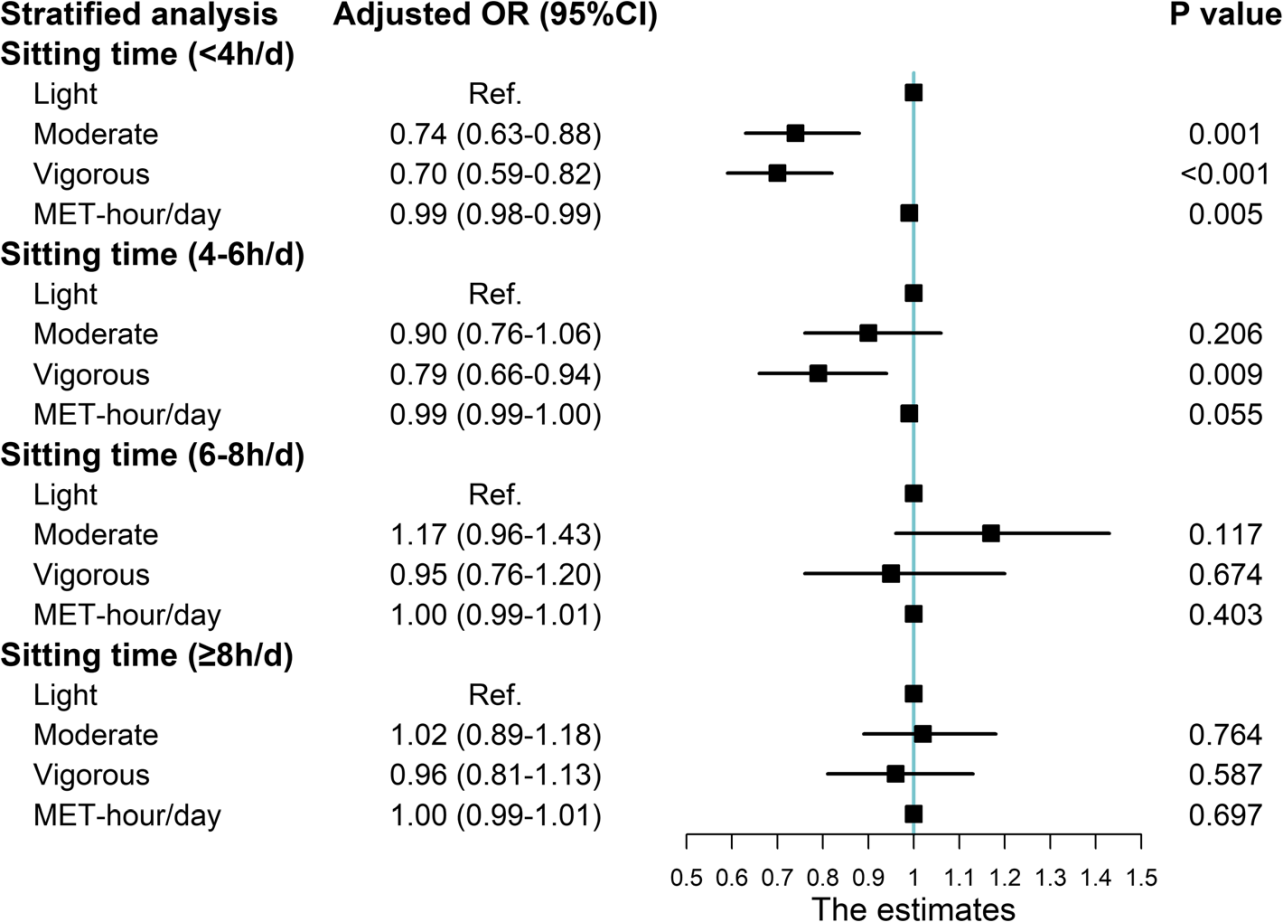
Stratified analysis for association of physical activity level with hyperuricemia by sitting time group. Adjusted for age, gender, education level, marital status, average monthly income, smoking status, drinking status, dietary pattern, and BMI

The results from stratified analysis by ST groups were presented in Fig. 2. After adjustment for confounders, only in ST < 4 h/d and 4–6 h/day groups, compared with the reference group (light PA), vigorous group had significantly decreased odds of T2DM. But in longer ST groups, no significant associations can be found (all *P* > 0.05). Meanwhile, in the joint analysis (Additional file 1: Table S3 and Figure S1), combinations of the light PA and the highest ST (≥ 8 h/d) were associated with a 75% increased risk for HUA compared with the reference group (OR: 1.75, 95% CI: 1.52, 2.01).

### Interaction of PA and ST on HUA

**Fig. 3:**
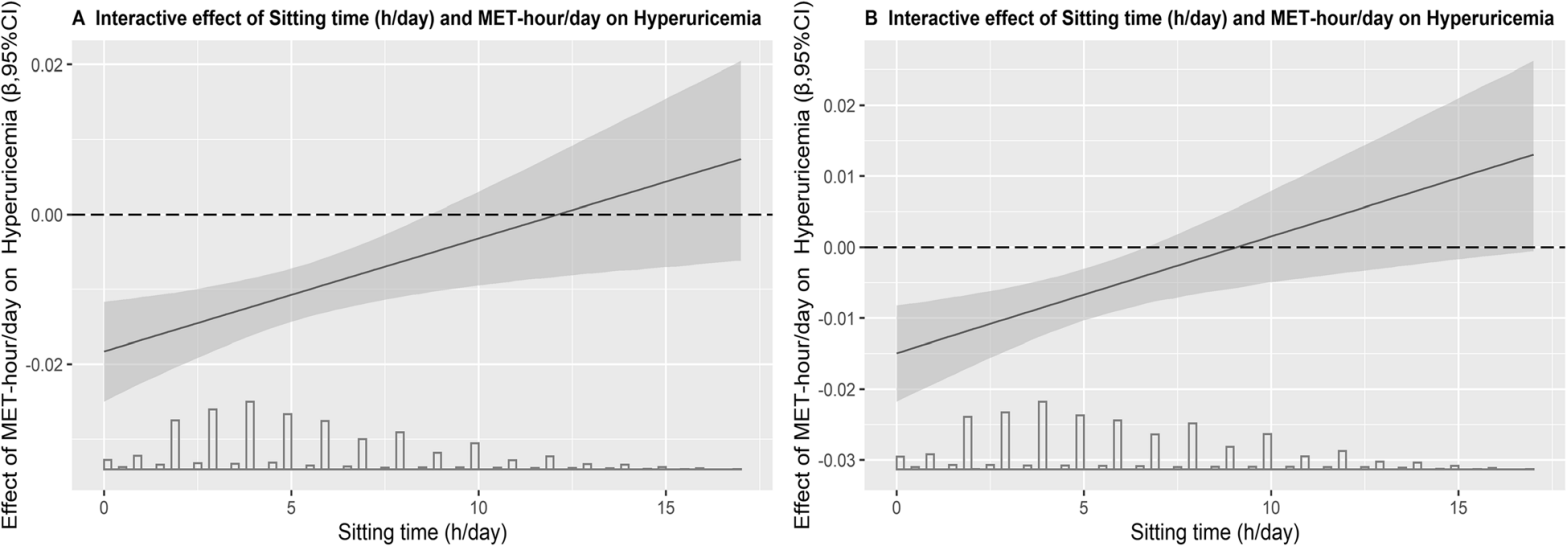
Regression estimates of physical effects (95% *CI*) on hyperuricemia as a function of sitting time by using generalized linear models. **a** Model 1: Adjusted for physical activity and sitting time level; **b** Model 2 included sitting time and physical activity as well as age, gender, education level, marital status, average monthly income, smoking status, drinking status, dietary pattern, and BMI

Furthermore, in order to explore the interaction of PA and ST, the effect of MET-hour/day on risk of HUA was plotted as a function of ST. Interaction plot was used to visualize the changes of effects of MET-hour/day on HUA along with increasing ST as shown in Fig. 3. In these models, the protective effects of PA-MET (hour/day) on HUA were attenuated with increasing ST (h/day).

## Discussion

In the current study, the results suggested that PA was independently associated with decreased serum uric acid level and prevalence of HUA, while ST was independently associated with increased serum uric acid level and prevalence of HUA. In addition, as the first study to examine the combined effect of PA and ST on HUA, we found the protective effects of PA-MET (hour/day) on HUA were weaken by increasing ST with a significant interaction effect (*P* < 0.001).

A lot of previous studies have found the protective effect of PA on metabolic health including various chronic metabolic diseases [23] and risk of mortality [24]. Despite health-enhancing benefits, PA alone may not be enough to reduce the risk of multiple chronic diseases [25]. Sedentary lifestyle, such as watching TV or computer has become more and more prevalent in modern society, and the total ST of a day has increased gradually in general population [26]. Emerging evidence suggests that prolonged ST was independently associated with increased risk of obesity, metabolic syndrome, T2DM, and CVD mortality after adjusting for PA [12]. However, the independent links among PA, ST, and HUA are not always well explored, and there are some inconsistent results. Several cross-sectional studies conducted to investigate the prevalence and lifestyle risk factors of HUA have explored the effect of PA on HUA [1, 8, 13]. A study from the rural Northeast Chinese population found that compared with the low PA, moderate, or high PA contributed to a lower prevalence of HUA in women, but not for men [1]. The results from the Chinese men and women show that PA-related lifestyle choices tend to have a beneficial effect, compared with exercise times > 6 times per week, exercise times 1–3 times, or < once per week significantly increased the serum uric acid level and risk of HUA [13]. The negative associations of PA with HUA between the current study and previous studies were consistent and the magnitudes of the associations could be comparable. But in a study conducted in the general Korean population, there have no found significant association between PA and HUA [8]. The racial differences of this association may need to be further explored. For the association of ST with HUA, Cui et al. reported that prolonged sitting was an independent risk factors of HUA [27]. In addition, only a recent study found that both regular PA and reduced sedentary time were independently associated with decreased prevalence of HUA [14], which was similar to the present results. Furthermore, our study found that combined light PA and the highest ST (≥ 8 h/d) may significantly increase the risk of HUA, which was also supported by some related findings [21].

Although the underlying mechanisms of HUA in relation to PA or ST remain unclear, several possible biological pathways have been suggested. First, PA can increase insulin sensitivity, which may be mediated the relationship between PA and serum uric acid level [28]. Second, regular physical exercise is recommended for reducing body weight, which is generally good for abnormal metabolic factors such as serum uric acid level, and further reducing the risk of various chronic diseases including HUA and CVD [29]. In addition, contrary to regular PA, prolonged ST may reduce insulin sensitivity, resulting in the increased serum uric acid level and risk of HUA [27].

An interesting finding in this study is that the negative association of PA with HUA was gradually attenuated by increasing ST with a significant interaction effect (*P* < 0.001). In addition, in the stratified analysis, when ST exceeded 6 h/d, the protective effect of PA on HUA became nonsignificant. To our knowledge, though there was no study explored for the interaction of PA and ST on HUA, several studies have assessed the interaction effect of PA and ST on metabolic health and risk of mortality and reported inconsistent findings [15, 30, 31]. One large-scale study examined the joint associations of PA and ST and found that the association between ST and all-cause mortality risk was gradually attenuated with increased PA [15]. But another study indicated that regular participation in high levels of moderate to vigorous PA does not fully protect against the risks of mortality associated with prolonged ST [30]. Moreover, similar to our finding, a cross-sectional study among Mexican Americans reported that moderate PA is beneficial for subclinical atherosclerosis in the low-level groups of ST (≤ 3 h/d), but there was no significant health effect when ST exceeded 3 h/day [31]. The exact mechanism of this interaction effect still remains unclear. Sitting too much may affect the cellular processes responsible for metabolic abnormalities differently than structured exercise as previously studied in the field of exercise physiology [32]. We speculated that excessive levels of ST may appear to contribute to higher odds for HUA regardless of PA level, which offset the beneficial effect of PA. Further studies are needed to clarify the potential mechanism.

The strengths of our study include its large-scale sample based on rural Chinese population, comprehensive statistical approach, and a series of standardized measures such as detailed assessment of PA, ST, and other potential covariates. Dose-response association between PA or ST and HUA was evaluated by restricted cubic splines, which was not found in other studies. In addition, this study is the first to investigate the interaction effect of PA and ST to gain a better understanding of how the combined effect of these variables on HUA.

Some limitations in the current study should be considered. First, this study only used the baseline survey data of the cohort, which cannot establish a cause-and-effect relation among PA, ST, and HUA, because of the cross-sectional study design. Second, demographic information and lifestyle characteristics including PA and ST were collected using a questionnaire; thus, recall bias may not be avoided. Third, although we have given for many potential confounders, it is likely that some residual confounding factors such as total energy intake might have affected the estimations. Unfortunately, these data are not available in the current study. Finally, we were unable examine the associations with different specific types of PA (aerobic or resistance exercise) and ST (watching TV/movies or transportation), and these detailed types are needed to be specified in future studies.

## Conclusions

In conclusion, this study in rural Chinese population demonstrates that regular PA may independently reduce serum uric acid level and prevalence of HUA, but prolonged ST may significantly increase serum uric acid level and HUA. In addition, excessive levels of ST (> 6 h/day) might offset the beneficial effect of PA on HUA. These findings suggest that both regular PA and reducing ST are important for preventing HUA. Moreover, interventions focused on reducing excessive ST may be a more effective strategy to decrease risk of HUA, which should be given more attention in public health.

## Supplementary Information

**Additional file 1: Table S1.** Association of physical activity level and sitting time with serum uric acid level by gender. **Table S2.** Association of physical activity level and sitting time with prevalence of hyperuricemia by gender. **Table S3.** Joint effects of hyperuricemia by different combinations of physical activity level and sitting time. **Figure S1.** Joint associations of physical activity level and sitting time with hyperuricemia.

## Data Availability

The datasets used during the current study are available from the corresponding author upon reasonable request. Please contact with Chongjian Wang about detail information. Email address: tjwcj2005@126.com

## Abbreviations

CVD: Cardiovascular disease
HUA: Hyperuricemia
PA: Physical activity
ST: Sitting time
MET: Metabolic equivalent
IPAQ: International Physical Activity Questionnaire
T2DM: Type 2 diabetes mellitus
CI: Confidence intervals
OR: Odds ratio

## References

[1] Singh G, Lingala B, Mithal A (2019). Gout and hyperuricaemia in the USA: prevalence and trends. Rheumatology (Oxford).

[2] Zhu Y, Pandya BJ, Choi HK (2011). Prevalence of gout and hyperuricemia in the US general population: the National Health and Nutrition Examination Survey 2007-2008. Arthritis Rheum.

[3] Drivelegka P, Forsblad-d'Elia H, Angerås O, Bergström G, Schmidt C, Jacobsson LTH (2020). Association between serum level of urate and subclinical atherosclerosis: results from the SCAPIS Pilot. Arthritis Res Ther.

[4] Dehghan A, van Hoek M, Sijbrands EJ, Hofman A, Witteman JC (2008). High serum uric acid as a novel risk factor for type 2 diabetes. Diabetes Care.

[5] Zhang H, Li Y, Mao Z, Liu X, Zhang X, Yang K (2018). Sex-specific associations of serum uric acid with metabolic syndrome in Chinese rural population: the RuralDiab study. Clin Chim Acta.

[6] Chang HY, Tung CW, Lee PH, Lei CC, Hsu YC, Chang HH (2010). Hyperuricemia as an independent risk factor of chronic kidney disease in middle-aged and elderly population. Am J Med Sci.

[7] Ting K, Gill TK, Keen H, Tucker GR, Hill CL (2016). Prevalence and associations of gout and hyperuricaemia: results from an Australian population-based study. Intern Med J.

[8] Kim Y, Kang J, Kim GT (2018). Prevalence of hyperuricemia and its associated factors in the general Korean population: an analysis of a population-based nationally representative sample. Clin Rheumatol.

[9] Chomistek AK, Manson JE, Stefanick ML, Lu B, Sands-Lincoln M, Going SB (2013). Relationship of sedentary behavior and physical activity to incident cardiovascular disease: results from the Women's Health Initiative. J Am Coll Cardiol.

[10] Krishnan S, Rosenberg L, Palmer JR (2009). Physical activity and television watching in relation to risk of type 2 diabetes: the Black Women’s Health Study. Am J Epidemiol.

[11] Sattelmair J, Pertman J, Ding EL, Kohl HW, Haskell W, Lee IM (2011). Dose response between physical activity and risk of coronary heart disease: a meta-analysis. Circulation.

[12] Owen N, Healy GN, Matthews CE, Dunstan DW (2010). Too much sitting: the population health science of sedentary behavior. Exerc Sport Sci Rev.

[13] Liu L, Lou S, Xu K, Meng Z, Zhang Q, Song K (2013). Relationship between lifestyle choices and hyperuricemia in Chinese men and women. Clin Rheumatol.

[14] Park DY, Kim YS, Ryu SH, Jin YS (2019). The association between sedentary behavior, physical activity and hyperuricemia. Vasc Health Risk Manag.

[15] Ekelund U, Steene-Johannessen J, Brown WJ, Fagerland MW, Owen N, Powell KE (2016). Does physical activity attenuate, or even eliminate, the detrimental association of sitting time with mortality? A harmonised meta-analysis of data from more than 1 million men and women. Lancet.

[16] Tu R, Li Y, Shen L, Yuan H, Mao Z, Liu X (2019). The prevalence and influencing factors of physical activity and sedentary behaviour in the rural population in China: the Henan Rural Cohort Study. BMJ Open.

[17] Dong X, Zhang H, Wang F, Liu X, Yang K, Tu R (2019). Epidemiology and prevalence of hyperuricemia among men and women in Chinese rural population: the Henan Rural Cohort Study. Mod Rheumatol.

[18] Liu X, Mao Z, Li Y, Wu W, Zhang X, Huo W (2019). Cohort Profile: The Henan Rural Cohort: a prospective study of chronic non-communicable diseases. Int J Epidemiol.

[19] Hou J, Liu X, Tu R, Dong X, Zhai Z, Mao Z (2020). Long-term exposure to ambient air pollution attenuated the association of physical activity with metabolic syndrome in rural Chinese adults: a cross-sectional study. Environ Int.

[20] Bennie JA, Chau JY, van der Ploeg HP, Stamatakis E, Do A, Bauman A (2013). The prevalence and correlates of sitting in European adults - a comparison of 32 Eurobarometer-participating countries. Int J Behav Nutr Phys Act.

[21] Stamatakis E, Gale J, Bauman A, Ekelund U, Hamer M, Ding D (2019). Sitting time, physical activity, and risk of mortality in adults. J Am Coll Cardiol.

[22] Wang B, Liu L, Qiao D, Xue Y, Liu X, Zhang D, et al. The association between frequency of away-from home meals and type 2 diabetes mellitus in rural Chinese adults: the Henan Rural Cohort Study. Eur J Nutr. 2020; 59(8):3815–25. 10.1007/s00394-020-02212-5 32193634

[23] Lear SA, Hu W, Rangarajan S, Gasevic D, Leong D, Iqbal R (2017). The effect of physical activity on mortality and cardiovascular disease in 130 000 people from 17 high-income, middle-income, and low-income countries: the PURE study. Lancet..

[24] Mok A, Khaw KT, Luben R, Wareham N, Brage S (2019). Physical activity trajectories and mortality: population based cohort study. BMJ.

[25] Biswas A, Oh PI, Faulkner GE, Bajaj RR, Silver MA, Mitchell MS (2015). Sedentary time and its association with risk for disease incidence, mortality, and hospitalization in adults: a systematic review and meta-analysis. Ann Intern Med.

[26] Lanningham-Foster L, Nysse LJ, Levine JA (2003). Labor saved, calories lost: the energetic impact of domestic labor-saving devices. Obes Res.

[27] Cui L, Meng L, Wang G, Yuan X, Li Z, Mu R (2017). Prevalence and risk factors of hyperuricemia: results of the Kailuan cohort study. Mod Rheumatol.

[28] Grandinetti A, Liu DM, Kaholokula JK (2015). Relationship of resting heart rate and physical activity with insulin sensitivity in a population-based survey. J Diabetes Metab Disord.

[29] Swift DL, Johannsen NM, Lavie CJ, Earnest CP, Church TS (2014). The role of exercise and physical activity in weight loss and maintenance. Prog Cardiovasc Dis.

[30] Matthews CE, George SM, Moore SC, Bowles HR, Blair A, Park Y (2012). Amount of time spent in sedentary behaviors and cause-specific mortality in US adults. Am J Clin Nutr.

[31] Walker TJ, Heredia NI, Lee M, Laing ST, Fisher-Hoch SP, McCormick JB (2019). The combined effect of physical activity and sedentary behavior on subclinical atherosclerosis: a cross-sectional study among Mexican Americans. BMC Public Health.

[32] Hamilton MT, Hamilton DG, Zderic TW (2007). Role of low energy expenditure and sitting in obesity, metabolic syndrome, type 2 diabetes, and cardiovascular disease. Diabetes.

