# Supplementary material for: Independent and interactive effect of sitting time and physical activity on prevalence of hyperuricemia: the Henan Rural Cohort Study
Source: Arthritis Res Ther. 2021 Jan 6;23:7. doi: 10.1186/s13075-020-02385-8 (PMC7789632; doi:10.1186/s13075-020-02385-8)

**Additional file 1:**

**Table S1** Association of physical activity level and sitting time with serum uric acid level by gender.

**Table S2** Association of physical activity level and sitting time with prevalence of hyperuricemia by gender.

**Table S3** Joint effects of hyperuricemia by different combinations of physical activity level and sitting time.

**Figure S1** Joint associations of physical activity level and sitting time and hyperuricemia. Adjusted for age, gender, education level, marital status, average monthly income, smoking status, drinking status, dietary pattern and BMI.

**Supplementary Table S1** Association of physical activity level and sitting time with serum uric acid level by gender.

|  | **Male (n=15371), *β* (95% *CI*)** | | **Female (n=23484), *β* (95% *CI*)** | |
| --- | --- | --- | --- | --- |
|  | **Model 1** | **Model 2** | **Model 1** | **Model 2** |
| **Physical activity level** |  |  |  |  |
| **Light** | 0 (Ref.) | 0 (Ref.) | 0 (Ref.) | 0 (Ref.) |
| **Moderate** | 0.70 (-2.63, -4.02) | 0.96 (-2.16, 4.08) | -1.59 (-3.64, 0.47) | 1.13 (-0.87, 3.13) |
| **Vigorous** | -7.48 (-10.63, 4.33) | -6.57 (-9.54, -3.60) | -4.68 (-7.01, 2.35) | -3.27 (-5.53, -1.01) |
| **MET-hour/d** | -0.48 (-0.60, -0.35) | -0.25 (-0.37, -0.13) | -0.13 (-0.22, -0.04) | -0.08 (-0.17, 0.01) |
| ***P* value for trend** | <0.001 | <0.001 | <0.001 | 0.002 |
| **Sitting time** |  |  |  |  |
| **<4h/d** | 0 (Ref.) | 0 (Ref.) | 0 (Ref.) | 0 (Ref.) |
| **4-6h/d** | 1.78 (-1.73, 5.29) | 2.50 (-0.79, 5.79) | 3.79 (1.57, 6.02) | 3.49 (1.34, 5.64) |
| **6-8h/d** | 5.46 (1.44, 9.48) | 8.53 (4.76, 12.29) | 8.72 (6.14, 11.30) | 8.63 (6.14, 11.13) |
| **≥8h/d** | 12.76 (9.26, 16.26) | 16.62 (13.34, 19.91) | 17.11 (14.77, 19.45) | 18.61 (16.34, 20.88) |
| **Per hour** **increased** | 1.30 (0.98, 1.69) | 1.90 (1.53, 2.27) | 2.18 (1.90, 2.45) | 2.31 (2.05, 2.58) |
| ***P* value for trend** | <0.001 | <0.001 | <0.001 | <0.001 |

Multivariable model 1 adjusted for physical activity and sitting time level where applicable; model 2 included sitting time and physical activity as well as age, education level, marital status, average monthly income, smoking status, drinking status, dietary pattern and BMI. *SD*, standard deviation; *CI,* confidence interval.

**Supplementary Table S2** Association of physical activity level and sitting time with prevalence of hyperuricemia by gender.

|  | **Male (n=15371), *OR* (95%*CI*)** | | **Female (n=23484), *OR* (95%*CI*)** | |
| --- | --- | --- | --- | --- |
|  | **Model 1** | **Model 2** | **Model 1** | **Model 2** |
| **Physical activity level** |  |  |  |  |
| **Light** | 1 (Ref.) | 1 (Ref.) | 1 (Ref.) | 1 (Ref.) |
| **Moderate** | 0.93 (0.82, 1.04) | 0.94 (0.83, 1.07) | 0.87 (0.78, 0.97) | 0.97 (0.87, 1.09) |
| **Vigorous** | 0.76 (0.68, 0.86) | 0.80 (0.71, 0.90) | 0.83 (0.73, 0.94) | 0.88 (0.77, 0.99) |
| **MET-hour/day** | 0.92 (0.90, 0.94) | 0.96 (0.93, 0.98) | 0.98 (0.96, 1.00) | 0.99 (0.97, 1.00) |
| ***P* value for trend** | <0.001 | <0.001 | 0.002 | 0.048 |
| **Sitting time** |  |  |  |  |
| **<4h/d** | 1 (Ref.) | 1 (Ref.) | 1 (Ref.) | 1 (Ref.) |
| **4-6h/d** | 1.08 (0.94, 1.23) | 1.11 (0.96, 1.27) | 1.16 (1.02, 1.32) | 1.14 (1.00, 1.29) |
| **6-8h/d** | 1.15 (0.99, 1.33) | 1.25 (1.07, 1.46) | 1.36 (1.18, 1.56) | 1.33 (1.15, 1.54) |
| **≥8h/d** | 1.22 (1.08, 1.39) | 1.38 (1.20, 1.58) | 1.61 (1.42, 1.82) | 1.64 (1.44, 1.86) |
| **Per hour increased** | 1.02 (1.01, 1.03) | 1.03 (1.02, 1.05) | 1.06 (1.05, 1.08) | 1.06 (1.05, 1.07) |
| ***P* value for trend** | <0.001 | <0.001 | <0.001 | <0.001 |

Multivariable model 1 adjusted for physical activity and sitting time level where applicable; model 2 included sitting time and physical activity as well as age, education level, marital status, average monthly income, smoking status, drinking status, dietary pattern and BMI. *OR, odds ratio; CI,* confidence interval.

**Supplementary Table S3** Joint effects of hyperuricemia by different combinations of physical activity level and sitting time.

| **Vigorous physical activity** | **Sitting time** | **Number** | ***OR* (95%*CI*)** |
| --- | --- | --- | --- |
|  | **<4h/d** | 4649 | 1 (Ref.) |
|  | **4-6h/d** | 3443 | 1.12 (0.96, 1.33) |
|  | **6-8h/d** | 1651 | 1.31 (1.08, 1.60) |
|  | **≥8h/d** | 1905 | 1.67 (1.40, 2.00) |
| **Moderate physical activity** |  |  |  |
|  | **<4h/d** | 3972 | 1.04 (0.88, 1.22) |
|  | **4-6h/d** | 4481 | 1.27 (1.09, 1.48) |
|  | **6-8h/d** | 3142 | 1.61 (1.38, 1.90) |
|  | **≥8h/d** | 3049 | 1.83 (1.56, 2.14) |
| **Light physical activity** |  |  |  |
|  | **<4h/d** | 3242 | 1.43 (1.23, 1.68) |
|  | **4-6h/d** | 2453 | 1.43 (1.21, 1.69) |
|  | **6-8h/d** | 1705 | 1.39 (1.15, 1.68) |
|  | **≥8h/d** | 5163 | 1.75 (1.52, 2.01) |

Adjusted for age, gender, education level, marital status, average monthly income, smoking status, drinking status, dietary pattern and BMI.

**Supplementary Figure S1** Joint associations of physical activity level and sitting time and hyperuricemia.


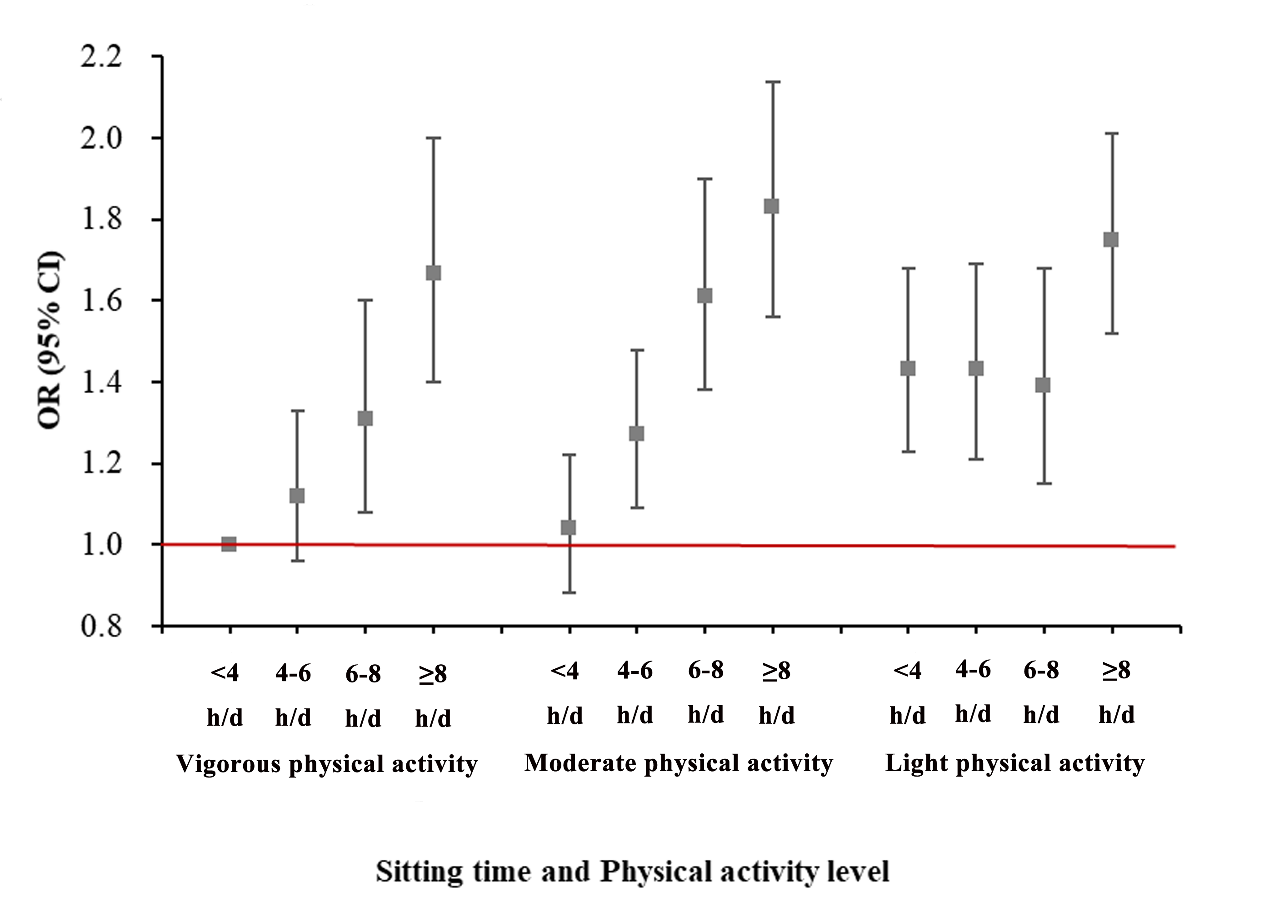

Supplement: Supplementary file 1 — Additional file 1: Table S1. Association of physical activity level and sitting time with serum uric acid level by gender. Table S2. Association of physical activity level and sitting time with prevalence of hyperuricemia by gender. Table S3. Joint effects of hyperuricemia by different combinations of physical activity level and sitting time. Figure S1. Joint associations of physical activity level and sitting time with hyperuricemia. [file 13075_2020_2385_MOESM1_ESM.docx]
